# Improving Calcium Knowledge and Intake in Young Adults Via Social Media and Text Messages: Randomized Controlled Trial

**DOI:** 10.2196/16499

**Published:** 2020-02-11

**Authors:** Anika Rouf, Monica Nour, Margaret Allman-Farinelli

**Affiliations:** 1 The University of Sydney School of Life and Environmental Sciences Camperdown Australia

**Keywords:** calcium, social media, young adults, randomized controlled trial, telemedicine

## Abstract

**Background:**

Calcium is an important nutrient for the attainment of peak bone mass during adolescence and young adulthood. However, these life phases are characterized as hard to reach for health promotion. Social media platforms offer a promising channel as they are relatively low cost but used ubiquitously by youth.

**Objective:**

The aim of the CAlcium Nutrition-Dietary Opportunities (CAN-DO) study was to conduct a randomized controlled trial to test the effectiveness of Facebook alone or with text messaging as channels to deliver a theory-based program to encourage optimal calcium intake.

**Methods:**

The intervention was a 3-arm parallel trial. Young adults aged 18 to 25 years were recruited through university and social media for a 6-week trial. Participants were randomized to 1 of the 3 arms (ie, Facebook posts, Facebook posts plus text messages, and control group that received an electronic leaflet containing information on calcium intake). The primary outcome was change in intake of milk and other calcium-rich foods, and secondary outcomes were knowledge, self-efficacy, motivation, and habit formation concerning calcium-rich foods. Changes were assessed before and after the intervention, and the differences in change between groups were compared using multivariate regression models with multiple imputations for missing data.

**Results:**

A total of 211 participants (64/211, 30.3% males) participated (mean age 21.4 years, SD 2.1) in this study. At the end of the program, no increase in milk intake (odds ratio [OR] 1.51, 95% CI 0.61-3.75 Facebook; OR 1.77, 95% CI 0.74-4.24 Facebook plus text messages; *P*=.41) nor calcium-rich food was detected (*P*=.57). There was a significant improvement in knowledge in the Facebook plus text messages group (*P*<.001), but habit formation improved less than that in the other 2 groups (*P*=.01). Our results showed a moderate level of engagement with intervention content and positive qualitative feedback from participants.

**Conclusions:**

The CAN-DO study delivered via Facebook (with the additional support of text messages) was found to improve knowledge and was acceptable among young adults. However, further research is needed to better understand social media engagement and how to optimize the program for participants to be sufficiently motivated to increase their intake of calcium-rich foods.

**Trial Registration:**

Australian New Zealand Clinical Trials Registry ACTRN12620000097943; http://www.anzctr.org.au/ACTRN12620000097943.aspx

## Introduction

### Background

As adolescents and young adults become increasingly independent, it is not uncommon for lifestyle behaviors to be adversely affected [1]. This may include decreased physical activity, increased rates of smoking and alcohol consumption, weight gain, and decreases in home-prepared meals [2-5]. Previous studies have shown that young adults are difficult to reach with traditional health promotion strategies [6,7], but it is important to support young adults through this transition to establish healthy dietary patterns for their own future health [3,8,9] and to potentially serve as role models to their children [10,11].

Among the consequences of poor-quality diets is a low intake of calcium, which remains a global concern [12] and among Australian young adults [13]. A secondary analysis from the most recent Australian National Nutrition Survey from 2011 to 2012 shows that 69% of males and 83% of females aged 19 to 25 years failed to meet the estimated average requirements for young adults [14]. An adequate intake of calcium in adolescence and young adulthood is important for the attainment of peak bone mass and prevention of osteoporosis in later life [15,16].

Our previous formative research has delved into the barriers and enablers to achieving adequate calcium intake for this population and revealed a gap in knowledge with respect to what amount of calcium-rich food constitutes a serve and the daily number of serves recommended [17]. Their level of motivation to improve calcium intake was low because a lack of knowledge meant more calcium seemed unnecessary, and financial factors influenced the opportunity to consume calcium-rich foods, wherein milk was seen as low cost, but sources such as nuts and fish were seen as high cost [17]. When asked about an appropriate medium to deliver an intervention program, the focus group participants preferred to learn from social media platforms, and Facebook was ranked as the preferred platform [17].

Young adults are ubiquitous users of social media [18]. Almost 90% of young adults (aged 18-29 years) access social media platforms at least once per day [19], so it has the potential for wide reach in an intervention. To date, the small evidence base for the effectiveness of nutrition-related interventions using a commercial social media platform, such as Facebook, is inconsistent and warrants further investigation [20-22]. Our previous meta-analysis of the effectiveness of interventions to increase calcium intake demonstrated a small effect size [23] but indicates that research into an intervention to improve calcium intake of Australian young adults is warranted.

### Objective

A previous Facebook intervention for weight loss in young adults found that the use of social media combined with text messages was effective for weight loss but not Facebook alone [20]. Previous electronic health interventions conducted in young adults found a high level of acceptability and engagement with text messages and effective dietary changes [24-26]. Therefore, the aim of this study was to determine the effectiveness of an educational and motivational program to improve calcium intake in young adults and whether the addition of text messages enhanced behavior change when compared with the Facebook arm alone.

## Methods

### Trial Design

This was a 3-arm parallel trial with a 1:1:1 allocation ratio. The 3 groups were Facebook intervention (Facebook), Facebook intervention plus text messages (Facebook plus text), and electronic leaflet (e-leaflet) containing information on calcium intake (control). The sample size was determined using G*Power (Version 3.1.9.4, Universität Kiel), a statistical power analysis software [27]. To detect a mean difference of 125 mg calcium intake with *P*=.05 and 90% power, assuming a standard deviation of 259 mg, a sample size of 45 was required per arm and increased to 75 to allow for 40% dropout.

### Participants

Young adults (males and females) aged 18 to 25 years were selected as this is the period where peak bone mass development is reached [28,29]. Inclusion criteria included owning a smartphone and a Facebook account. Exclusion criterion was having completed a nutrition course or currently undertaking a nutrition course on the basis of their high existing level of nutrition knowledge. In addition, any participants with a food allergy, known lactose intolerance, or currently taking calcium supplements (but not multivitamins) or eating disorders were excluded.

All materials and methods of the intervention were approved by the Human Research Ethics Committee at the University of Sydney, Australia. The ethics approval number is 2018/597. Each participant was reimbursed with an Aus $10 voucher after completing the final questionnaire. This offer did not impact the voluntary nature of consent as it was provided after the intervention finished rather than at the time of consent. The reporting of outcomes was guided by the Consolidated Standards of Reporting Trials of Electronic and Mobile HEalth Applications and onLine TeleHealth checklist [30]. As neither the primary outcome nor the secondary outcomes were clinical measurements, the study was not entered into a clinical trials registry.

### Randomization and Concealment

A randomized sequence generation was used to allocate the participants. The randomization was performed by 2 independent researchers who were not study investigators.

### Recruitment

Recruitment strategies included social media (posts to friends and paid advertising on Facebook), posting on University website (*volunteer for research study*), flyers (on campus noticeboards), volunteers on a research database (previous volunteers who took part in nutrition research and agreed for contact in the future), and active face-to-face recruitment. For each of the abovementioned recruitment methods, the potential participant was made aware that participation was voluntary. Interested participants accessed the screening questionnaire for eligibility before joining the study.

### CAlcium Nutrition-Dietary Opportunities Program

A theory-informed step-wise approach was used to develop the CAlcium Nutrition-Dietary Opportunities (CAN-DO) program using the Behavior Change Wheel system [31]. This framework posits that an individual requires capability (C), opportunity (O), and motivation (M) to perform a certain behavior (B) and includes a series of 9 intervention functions that can be mapped to the COM-B components [31,32].

The aim was to build relevant knowledge (capability) and influence beliefs and attitudes to generate intentions for individuals to change behaviors (reflective motivation). The details of the intervention functions and relevant behavior change techniques are presented in Table 1. In brief, the behavior change techniques included goal setting (behavior), self-monitoring of behavior, social support (unspecified), instruction on how to perform a behavior, information about health consequences, behavior substitution, habit formation, credible source, and restructuring the physical environment.

**Figure 1:**
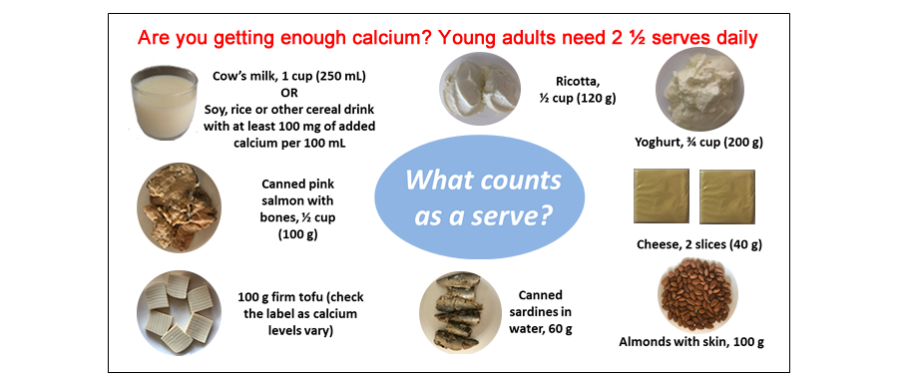
Infographic to inform participants of the recommendations.

The content of the intervention was developed in 2 parts. A range of instructional videos was created to build skills in cooking calcium-rich, low-cost, and mostly plant-based meals. These were tested in focus groups for acceptability and refined based on the feedback (unpublished findings). The next step was to design text messages and Facebook posts tailored to the preferences of young adults as indicated in prior formative research [33]. The intervention content was focused on educating on calcium-containing food sources and recommended serves, tips for including more calcium, and recipe videos that provided instructions on how to incorporate calcium in main meals and snacks. Text messages were kept short (<160 characters) and designed to complement the Facebook posts. Text messages and Facebook posts also reminded participants about setting goals and tracking progress for habit formation and created social support via posts and 2-way text messaging. An infographic was created to inform participants of the recommendations and set as a cover photo on the Facebook page (Figure 1). The e-leaflet that was provided to participants in the control group is shown in Multimedia Appendix 1.

**Table 1:** Details of behavior change techniques with an example.

Intervention function	BCT^a,b^ code	Name of BCT	An example of a Facebook post	An example of a text message
Enablement	1.1	Goal setting (behavior)	Males and females aged between 18 to 30 years should aim to consume about 1000 mg or 2.5 serves of dairy and/or alternatives per day. How much are you having? Check out this infographic which shows examples of what counts as a serve and set yourself a goal to have one more serve per day.	Hi [insert name], it’s Anika from the CAN-DO^c^ program. It’s time to set your goals and start tracking! Have you downloaded your app and set a goal? Please reply to this message by typing YES or NO.
Enablement	2.3	Self-monitoring of behavior	Calcium intake is low in the Australian population; 44% of males and 71% of females aged 18 to 30 years don’t get enough. Monitoring your progress can be useful when trying to establish new habits. You can use the app “Productive” (for iPhones) and “Loop Habit Tracker” (for Android) to track your intake.	Hi [insert name], are you still using the app to track your goals? Please reply to this message by typing YES or NO.
Enablement	3.1	Social support (unspecified)	Have you tried tofu? It is a great alternative to eggs and can be scrambled together with leftover veggies for breakfast. PS: Do you have any breakfast ideas you’d like us to share? Let us know what recipes you have tried in the comments below:)	Hi [insert name], it’s Anika from the CAN-DO program. Did you check out the Facebook post yesterday? Give us a thumbs up if you like it.
Training	4.1	Instruction on how to perform a behavior	Not only is fish great for heart health, but some varieties are a good source of calcium. You can opt for canned options such as salmon or sardines that will save you time and money. Check out this salmon cannelloni recipe for a delicious way to cook with canned fish.	Hi [insert name], Have you tried any of the recipes from the cooking videos we’ve shared so far? Please reply to this message by typing YES or NO.
Education	5.1	Information about health consequences	It is important to get your calcium everyday as it can lower your risk of chronic diseases. Here’s a photo of a veggie platter I created recently. I used the Tzatziki recipe shared on Monday as a side dip to boost the flavor. Make sure you give this a go and share your veggie platter with us :)	Hi [insert name], Did you know that calcium is important for your bone strength? To up the calcium, why not try anchovies and vegetables on your pizza.
Enablement	8.2	Behavior substitution	Is takeaway your go-to for work lunch? Try cooking larger amounts at dinner and taking the leftovers the next day for a healthier alternative. These delicious stuffed capsicums contain ricotta and parmesan and taste even better the next day. You can even use canned salmon to bump up the calcium content.	Hi [insert name], Do you get afternoon munchies? Why not swap those chips with some wholegrain crackers and cheese? Cheese is a great source of calcium and protein, and will help you beat the 3 pm slump.
Training	8.3	Habit formation	Research has shown that eating breakfast improves your cognitive function and memory. If you are not a breakfast eater, it’s time to change and look after yourself! Here’s an overnight chia pudding recipe for you to try.	Hi [insert name], How much calcium are you having now? Even if you’ve only increased a little, WELL DONE! You’re on your way to healthier habits.
Persuasion	9.1	Credible source	Did you know that low fat dairy products have just as much as calcium as regular varieties? The Australian Dietary Guidelines advise that more than 50% of intake from dairy foods should be reduced-fat varieties. Check out this infographic!	Hi [insert name], Research shows that having calcium at breakfast increases your chance of meeting your requirement. Did you have your breakfast today? Reply YES or NO.
Environmental restructuring	12.1	Restructuring the physical environment	Need some meal prep inspiration? Here is a Mac and Cheese recipe you could try at home. Having pre-prepared meals in your fridge will help you avoid the temptation of take-away and keep you on track with healthy eating. TIP: to save time, you can use multiple containers to store so it is ready to grab and go for the next day!	Hi [insert name], Some canned varieties of fish with bones like salmon and sardines are a great source of calcium. Stock your pantry with canned fish for a quick calcium-rich sandwich filler.

^a^BCT: behavior change technique.

^b^BCTs were derived from Behavior Change Technique Taxonomy (version 1).

^c^CAN-DO: CAlcium Nutrition-Dietary Opportunities.

### Procedures

**Figure 2:**
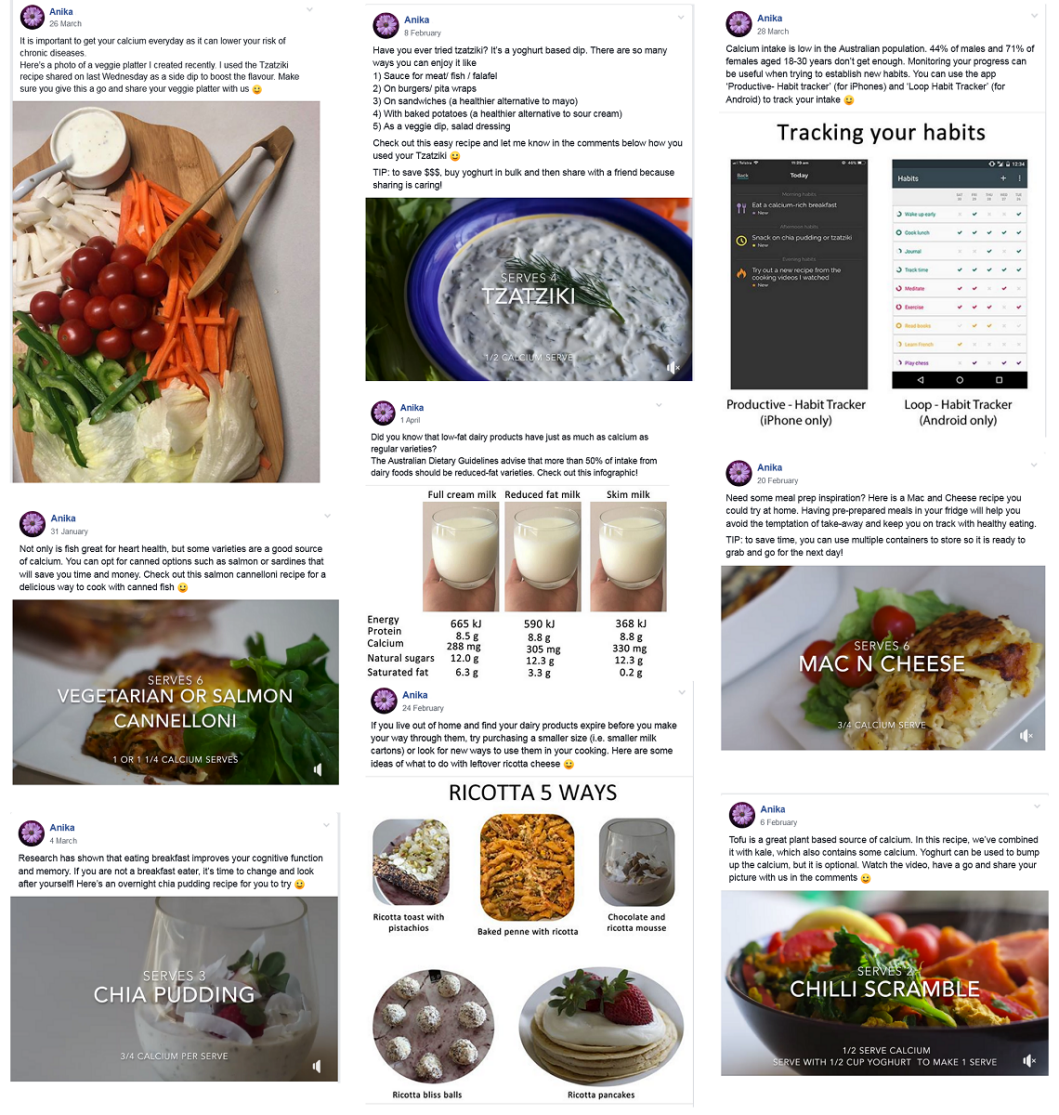
Screenshots displaying Facebook posts that included photos, videos and the tracking apps for goal-setting and self-monitoring.

Interested participants completed a screening questionnaire hosted on Research Electronic Data Capture (REDCap) [34], where they could find out more about the study by reading the participant information statement and check their eligibility. Participants who were not eligible to participate were provided with the Australian Dietary Guidelines as a resource. Eligible participants completed the consent form and proceeded to the baseline questionnaire. After completing the baseline questionnaire, each participant was randomized to Facebook, Facebook plus text, or control group and received an email with their group allocation. Participants in the Facebook and Facebook plus text groups were invited to join a closed Facebook group, where a post was made every alternate day by the researcher (AR). The site had a pinned Facebook post used to ensure that all participants were provided with background information, which included links to educational resources and an overview of the intervention. Screenshots of the posts are shown in Figure 2. The 2 Facebook groups were kept separate to avoid potential contamination between groups. In addition, participants in Facebook plus text group were sent text messages every alternate day to the post. Participants in both intervention groups were encouraged to set goals using apps available on iPhone (Productive—Habit Tracker) and Android platforms (Loop—Habit Tracker) and self-monitor their progress. The participants in the control group were emailed once with an e-leaflet on calcium and did not receive any continued support on social media. This minimal intervention was to maintain their interest in completing the study.

### Measurement of Outcomes

Demographic information was collected from all participants, which included age, gender, educational level, postcode (for categorizing socioeconomic status), occupation, and income through a Web-based platform REDCap [34]. The postcode was used to categorize the socioeconomic status of participants using Socio-Economic Indexes For Areas (SEIFA) [35]. All outcomes were assessed at 2 time points, which were at baseline before commencing the study (T0) and at the end of intervention (T1) via a Web-based questionnaire on REDCap.

The primary outcome (calcium intake) was estimated using a validated calcium-specific food frequency questionnaire that asks about intake over the past week [36]. Milk was measured in cups, ranging from half a cup to more than 4 cups, and the other calcium-rich foods (30 foods and beverages included) were measured by weekly frequency of intake only. The secondary outcomes measured the impact of the intervention on determinants of calcium intake, which included knowledge of calcium recommendations and serve sizes, self-efficacy, motivation for consuming calcium-rich foods, and habit formation. Knowledge was assessed by a participant’s ability to identify sources of calcium (maximum of 8), a correct serve of calcium (maximum of 8), and stating the calcium requirements for their age group (maximum of 2) using a researcher-designed questionnaire as no validated questionnaire could be found. The questions are included in Multimedia Appendix 2. A 5-item Likert scale questionnaire previously validated for other dietary behaviors was adapted to measure self-efficacy for improving calcium-rich food consumption. The maximum score possible was 25; a higher score indicated stronger self-efficacy [37]. Autonomous and controlled motivation for consumption of calcium was measured using a 4-point scale. The questions were adapted from the Self-Regulation Questionnaire [38,39], where a higher score indicated greater motivation (score out of 16). Habit formation for calcium intake was measured using the validated 4-item 7-point scale Self-Report Behavioral Automaticity Index [40]. A greater score indicated a higher automaticity to perform a certain behavior.

### Engagement and Process Evaluation

Engagement with the platform was measured quantitatively and qualitatively as research indicates the need to do both [41]. Quantitative measures were obtained from recording Facebook analytics. After all participants had completed the intervention, the number of participants who had seen, liked, and commented on the Facebook posts was recorded. For the Facebook plus text group, the number of replies to text messages was counted for each participant, and the content was analyzed using qualitative methods (see Qualitative Analyses below).

Feedback regarding the acceptability of the program was collected via open-response questions regarding ease of use, usefulness of program, likelihood of recommendation to others, and overall enjoyment using Likert scales (5 being highest). The other optional questions were related to intervention experience and uptake of content as well as frequency and reason for engagement. The last question provided participants with an opportunity for free text comments.

### Statistical Analysis

To account for all participants, an intention-to-treat analysis with multiple imputations for missing values was used. This meant that all participants who were randomized at the start of the trial were retained for analyses. Owing to the large amount of missing data, 10 imputed datasets were created based on gender, SEIFA (socioeconomic index), cooking frequency per week, and baseline intake of primary (milk and calcium intake) and secondary (knowledge, self-efficacy, motivation, and habit) outcomes using Stata version 13.1 (StataCorp LP).

The primary outcome of change in milk intake, which was categorical in number of cups, was compared between 3 groups using a logistic regression model adjusted for gender, SEIFA, cooking frequency, baseline calcium (nonmilk), baseline knowledge, baseline self-efficacy, baseline motivation, and baseline habit. The quantitative values for change in calcium intake from other dietary sources were compared using linear regression as were the variables for the secondary outcomes of knowledge score, self-efficacy for change score, and motivation and habit score, adjusted for gender, SEIFA, cooking frequency, baseline calcium intake, baseline knowledge, self-efficacy, motivation, and habit. An analysis using completers-only data was conducted and is available in the Multimedia Appendices 3-5. The distribution of missing outcome data at both time points was investigated using counts and percentages across all sociodemographic variables. Furthermore, separate general estimating equation (GEE) models for binary data were used to investigate any relationships between sociodemographic variables and missingness in each outcome, adjusted for other sociodemographic variables. An independent samples *t* test was used to assess differences in number of views, likes, and comments for Facebook posts between the 2 groups receiving the intervention (SPSS for Windows 22.0 software IBM Corp, released 2013). A *P* value of less than .05 was considered statistically significant for all tests.

### Qualitative Analyses

The feedback from the final questionnaire was transcribed and analyzed using an inductive approach where common themes were grouped together. The NVivo 12 Plus (2018, version 12.2.0; QSR International Pty Ltd) software was used for thematic analyses.

## Results

### Participant Characteristics

**Figure 3:**
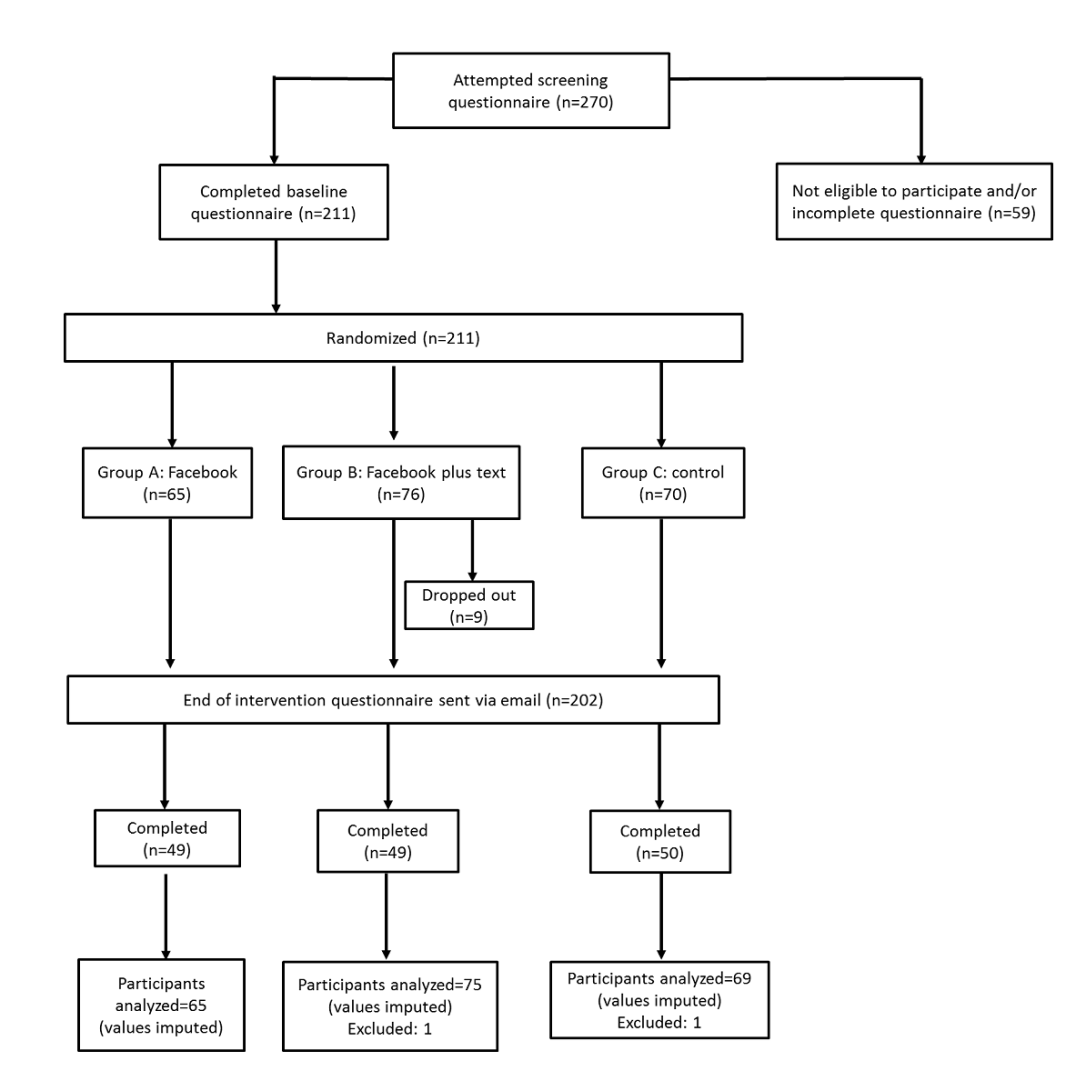
Participant flow diagram in the CAlcium Nutrition-Dietary Opportunities study.

A total of 270 participants attempted the screener questionnaire. Of 270 participants, 59 were ineligible for the study or failed to continue to the baseline questionnaire. A total of 211 young adults were randomized into 3 groups. The flow of participants through the trial is shown in Figure 3. The characteristics and demographics of participants at baseline are presented in Table 2. The mean age was 21.4 years (SD 2.1), and the sample comprised 30.3% (64/211) males.

The majority of participants (139/211, 65.9%) were enrolled in tertiary education. Nearly one-third (65/211, 30.8%) of the participants were in health care for their field of work or study. Almost two-thirds (134/211, 63.5%) of the participants were earning less than Aus $500 per week. Nearly half (94/211, 44.5%) of the sample reported themselves as being the main purchaser of household groceries. The most commonly reported cooking frequency was less than twice weekly for 37.4% (79/211) of the young adults.

**Table 2:** Demographics of participants from the CAlcium Nutrition-Dietary Opportunities study.

Baseline characteristics		Facebook (n=65)	Facebook plus text (n=76)	Control (n=70)
Age (years), mean (SD)		21.3 (2.2)	21.6 (2.0)	21.4 (2.1)
**Gender (n)**				
	Male	22	24	18
	Female	43	52	52
**Occupation (n)**				
	Student	38	53	48
	Full-time work	15	14	15
	Part-time or casual work	10	5	6
	Unemployed	2	4	1
**Field of work or study (n)**				
	Health care	22	20	23
	Management or finance	5	7	0
	Other^a^	38	49	47
**Highest level of education (n)**				
	≤Year 12	35	34	37
	Certificate or diploma	14	14	13
	Bachelor or postgraduate degree	16	27	20
	Prefer not to say	0	1	0
**Socio-Economic Indexes For Areas** **(n)**				
	Quintiles 1 and 2	20	25	27
	Quintile 3	15	29	19
	Quintiles 4 and 5	30	21	23
**Income per week^b^ (n)**				
	Nil or negative income	8	19	12
	Aus $1-Aus $499 per week (Aus $1-Aus $25,999 per year)	28	32	35
	Aus $500-Aus $999 per week (Aus $26,000-Aus $51,999 per year)	18	15	13
	More than Aus $500 per week	11	10	10
**Purchaser of main household groceries (n)**				
	Myself	36	51	7
	Others (partner, parents, and housemate/s)	29	25	63
**Cooking frequency per week (n)**				
	Less than twice a week	23	29	27
	3-4 times per week	24	24	26
	5 or more times per week	18	23	17
Rating of own cooking skills (0-100), mean (SD)		63.0 (22.4)	59.4 (21.6)	57.6 (22.2)

^a^Some options from the other field of study or work include education, office support and management, food service industry, information technology, and building or construction.

^b^Includes wages/salaries, government benefits, allowances, and other income, excluding tax, superannuation contributions, or any other automatic deductions.

### Attrition

Overall, 9 participants formally withdrew from the study. All participants were from the same arm (Facebook plus text) and opted out by sending a text—an option not available to other participants who could only opt out passively. The dropout time ranged from day 1 to day 29. Only 2 participants provided reasons (ie, lack of interest or time). In total, 148 (148/211, 70.1%) participants completed the final questionnaire but not necessarily every question.

### Outcomes

Results from 209 participants (data from 2 participants could not be imputed because of incorrect postcodes) are reported in the following sections. Results using completers-only data are included in Multimedia Appendices 3-5. The percentage of data that were missing was approximately 35% for milk intake, knowledge, self-efficacy, motivation, and habit, but 75% for calcium-rich foods. This percentage was similar across all sociodemographic variables. The GEE indicated that females had lower adjusted odds ratio (OR) than males of have missing data, adjusted for all other sociodemographic variables. No other sociodemographic variables were associated with missing outcome values.

#### Primary Outcomes

Participants in the Facebook group were 1.51 times more likely to move to a higher milk category compared with those in the control group (Table 3). Similarly, those in the Facebook plus text group were 1.77 times more likely to move to a higher milk intake category. However, this was not significant (*P*=.41). There was no difference in the change in calcium intake from other foods between groups over the 6 weeks (*P*=.57; Table 4). The analysis on completers-only data demonstrated a significant increase in milk intake in the Facebook plus text messages group compared with the control group (OR 4.99, 95% CI 1.63-15.28).

**Table 3:** Change in category of the amount of milk intake from baseline to the end of the intervention for all participants (n=209, using imputed dataset); overall *P*=.41.

Groups	Baseline milk intake (%)			Participants who moved to a higher milk intake category (%)	Percentage increase (95% CI)	Odds ratio of participants moving to a higher milk intake category (95% CI)^a^	*P* value
	<125 mL	125-249 mL	>250 mL				
Facebook (n=65)	38.0	36.2	25.9	35.8	22.1-49.6	1.51 (0.61-3.75)	.37^b^
Facebook plus text (n=75)	27.7	40.0	32.3	41.2	29.0-53.4	1.77 (0.74-4.24)	.20^b^
Control (n=69)	37.1	30.3	32.2	28.1	15.8-40.4	Reference	Reference

^a^Covariates appearing in the logistic regression model have been adjusted for gender, Socio-Economic Indexes For Areas, cooking frequency, baseline calcium (except milk) intake, baseline knowledge, baseline habit, baseline motivation, and baseline self-efficacy. The logistic regression model was not adjusted for baseline milk consumption because everyone in the lower category having to stay the same or increase or everyone in the higher category having to stay the same or decrease. This resulted in a zero-cell count for these baseline categories in the respective outcome (0=same or decrease and 1=increase).

^b^*P* value is comparison with control as a reference.

**Table 4:** Change in calcium intake per day in mg (excluding milk) from baseline to the end of the intervention using logistic regression for all participants (n=209, using imputed dataset); *P*=.57.

Groups	Mean baseline intake (SE)	Mean change^a^ (SE)
Facebook (n=65)	234.3 (25.0)	7.1 (39.1)
Facebook plus text (n=75)	271.9 (33.5)	65.5 (48.4)
Control (n=69)	226.4 (26.8)	43.0 (30.7)

^a^Covariates appearing in the linear regression model have been adjusted for gender, Socio-Economic Indexes For Areas, cooking frequency, baseline milk intake, baseline calcium intake, baseline knowledge, baseline habit, baseline motivation, and baseline self-efficacy.

#### Secondary Outcomes

Changes in secondary outcomes are reported in Table 5. The answers to the knowledge questions were combined together as an overall knowledge score. The change in knowledge was significant between the groups (*P*<.001). Those in the Facebook plus text intervention arm had a greater improvement in mean score compared with those in the Facebook and control groups. No significant difference between groups was observed for motivation (*P*=.79) or self-efficacy (*P*=.31). For habit formation, a significant group effect was observed (*P*=.01), with Facebook plus text group having the least increase in score. The improvement in knowledge in the Facebook plus text messages group was also found with completers-only analysis (*P*=.04). The effect on habit formation was not shown in the completers-only analysis.

**Table 5:** Change in secondary outcomes from baseline to the end of the intervention for all participants (n=209, using imputed dataset).

Outcome	Facebook		Facebook plus text		Control		*P* value^a^
	Mean baseline value (SE)	Mean change (95% CI)	Mean baseline value (SE)	Mean change (95% CI)	Mean baseline value (SE)	Mean change (95% CI)	
Habit formation score (out of 28)	15.7 (0.7)	3.5 (1.6 to 5.3)	16.4 (0.8)	0.5 (0.93 to 2.0)	15.3 (0.8)	3.4 (1.7 to 5.2)	.01
Overall knowledge score (out of 18)	6.7 (0.3)	1.6 (0.6 to 2.5)	6.3 (0.3)	2.9 (2.0 to 3.8)	6.6 (0.3)	0.2 (−0.7 to 1.1)	<.001
Motivation score (out of 16)	10.4 (0.4)	1.0 (0.3 to 1.7)	10.4 (0.3)	1.0 (0.3 to 1.8)	10.5 (0.3)	1.1 (0.4 to 1.9)	.79
Self-efficacy (out of 25)	19.4 (0.6)	1.2 (−0.1 to 2.4)	19.5 (0.5)	0.5 (−0.8 to 1.8)	17.8 (0.6)	1.0 (−0.4 to 2.4)	.31

^a^Covariates appearing in the linear regression model have been adjusted for gender, Socio-Economic Indexes For Areas, cooking frequency, baseline milk intake, baseline calcium intake, baseline knowledge, baseline habit, baseline motivation, and baseline self-efficacy.

### Engagement

#### Facebook Posts and Text Messages

Table 6 shows the engagement with Facebook posts. More participants in the Facebook plus text intervention than those in the Facebook intervention viewed the posts and liked them (*P*<.001 for both). In the Facebook group, 3 participants made comments on posts, whereas 4 participants in the Facebook plus text group commented on posts.

For the Facebook plus text group, the mean number of replies from participants was 3.8 out of a maximum 21 (range 1-18). Of 75 participants, 12 made no reply texts (1 participant gave a wrong phone number, and texts could not be delivered). The highest number of replies was to the yes/no response as to whether they had set a goal on the app (n=22).

**Table 6:** Engagement with the program on Facebook.

Engagement recorded on Facebook per post	Facebook, mean (SD)	Facebook plus text, mean (SD)	*P* value^a^
Seen by	19.9 (3.6)	26.9 (5.0)	<.001
Likes	1.1 (1.4)	3.6 (2.4)	<.001
Comments	0.1 (0.5)	0.2 (0.9)	.41

^a^Conducted using an independent samples *t* test.

### Process Evaluation

The majority of participants (n=133) completed the process evaluation questions, and Table 7 shows that there were no differences between intervention groups as to ease of use, their liking, likelihood of recommending it to others, or usefulness of the program. Participant responses in relation to message reading and interactions are included in Multimedia Appendix 6.

The thematic analysis with representative quotes is tabulated in Multimedia Appendix 7. The themes were grouped into ease of use, raised awareness, increased intake, feedback on recipes, reasons for reading/posting, and suggestions for improvement. Any comments that did not fit into these 5 groups were labeled as *general feedback*. There was a divergence of opinion on the ease of use, with some participants suggesting it was easy to follow, and others had more difficulty understanding and wanted more feedback. Successful participants shared their accomplishments in achieving their goals. The feedback on the recipes was overall positive, but some participants admitted they never prepared any of them. The majority of respondents chose not to share posts with reasons being they were uncertain they could add anything extra to the conversation or they did not feel comfortable with sharing. Some of the suggestions for improvement under general feedback included using an alternate platform that allows for active chat between members, sending text messages more frequently to check up on their progress, completing surveys weekly to track progress, and organizing meetings in person. Most participants viewed the notifications as a gentle and helpful reminder, whereas some found it intrusive.

**Table 7:** Process evaluation of the CAlcium Nutrition-Dietary Opportunities study on intervention experience.

Questions asked^a^	Facebook (n=45), rating (mean [SD])	Facebook plus text (n=44), rating (mean [SD])	Control (n=44), rating (mean [SD])
How easy was it to follow the program?	3.80 (0.89)	3.73 (1.25)	4.0 (0.96)
How much did you like the program?	3.54 (0.89)	3.57 (1.15)	3.82 (0.92)
How likely are you to recommend it to others?	3.41 (1.03)	3.50 (1.25)	3.80 (1.02)
How useful was the program to you?	3.35 (0.98)	3.57 (1.11)	3.57 (1.10)

^a^Participants were asked to rate on a scale of 1 to 5 (5 being highest).

## Discussion

### Principal Findings

This study showed that a 6-week intervention to increase calcium intakes tailored to young adults delivered using a social media platform and text messages was successful in improving knowledge about calcium-rich foods. However, this did not result in a significant increase in calcium-rich food and beverage intakes. The Facebook intervention delivered alone failed to show knowledge improvement, but engagement with the social media was significantly less than that in the intervention arm receiving text messages and might explain the disparate finding. Other reasons for the difference might be that the additions of texts appear to provide a more personalized program, and the need to reply to some messages engenders accountability and perception of monitoring by the staff delivering the intervention.

The findings of a successful outcome from the combined intervention arm concur with the earlier findings of a weight loss program delivered to overweight and obese college-aged students. Over 8 weeks, topics (1 per week) about weight loss were posted on Facebook, and the other intervention arm additionally received text messages with personalized feedback each week [20]. Although our text messages were generic, the participants’ names were included, and they were written in the Generation Y tone for which young adults had previously expressed a preference [33]. The texts provided additional prompts to set goals and self-monitor their own behavior with some further education and persuasion. These 2 behavior change techniques have been demonstrated to result in behavior change [42].

Few dietary changes occur as a result of education alone, but it was indicated as a necessary antecedent to behavior change in this demographic based on our previous focus group findings. Although the Facebook plus text group improved knowledge, the overall score remained quite low, with the mean score only reaching 50% correct answers. Another barrier to improving calcium intakes seemed to stem from lack of motivation, with all groups scoring similarly at baseline (10 out of 16), with uniform small improvement at the end of the intervention. In future programs, more planning around the inclusion of other techniques to improve reflective motivation may be needed. Coercion, persuasion, and incentivization could be possible solutions [31]. Social media platforms readily offer the capacity for monitoring of an individual’s behavior by others, and social comparison could be applied to intake of calcium-rich foods in this case. The vacillation might be that members are uncomfortable with sharing information with others as seen here in the replies to the process evaluation. The lack of posts made by group members is also indicative that such an approach may not work to positively influence motivation. Further research to understand what would allow participants to be relaxed with sharing dietary information in a nutrition intervention is desirable. With regard to incentivization and rewards, an earlier qualitative study with young adults for the co-design of an intervention to improve vegetable intakes reported that self-rewards were unlikely to motivate them as it required too much self-organization, so social or material rewards may be a better choice [21].

The validity of the food frequency instrument to measure changes in the primary outcome of calcium from milk and other foods in this population must also be questioned. Any self-report tool is always subject to participant bias [43]. In addition, this tool may not possess sufficient precision to detect small changes in intakes, as milk intakes are categorized in cups from half a serve of dairy to 4 or more serves of dairy. The calcium-specific food frequency questionnaire was selected rather than other tools as the burden of completion was low, but it does serve to rank individuals rather than assess absolute intake, and hence, the OR of increasing category of intake was used here.

Improvements in calcium intake were not achieved, but the retention and engagement in the social media intervention were substantial for an electronically delivered intervention [44]. Overall, 70% of the sample was retained, and more than half of the participants viewed the posts. Previous studies report large attrition and declining engagement in social media interventions for improving health behaviors [45]. A strength of the CAN-DO study was the formative research conducted to inform program design and materials [33]. The components were generally well received, and the recipe videos commended.

Among the limitations of this study was the overrepresentation of females comprising 70%, but this is not uncommon in nutrition studies even when males are equally targeted. In addition, in the case of calcium, it is females who are more likely to have inadequate intakes, so the population participating was appropriate. Some participants who did not do their own grocery shopping and cooked infrequently may have lessened the opportunity to alter their meals and snacks. The length of the program may have been too short to see the changes in knowledge translate into changes in consumption of calcium-containing foods, and intakes were only measured at 2 time points. An intervention delivered to university students that included a face-to-face session followed by text messages for 10 weeks did show increases in calcium intake [46]. In the future, a longer intervention might be appropriate. Finally, to include the largest number of participants, multiple imputation was used. This increases the variance in the estimate and a more conservative interpretation of results than completers-only analysis.

### Conclusions

The CAN-DO study was found to be feasible to deliver in our selected target population. Our qualitative results from the process evaluation mostly indicate that participants enjoyed the program. However, the quantitative result shows that 1 (change in knowledge) out of 5 outcomes improved in the Facebook plus text messages group only. The CAN-DO study has provided valuable insights into the process of disseminating a social media intervention for young adults, and a number of changes to program design are indicated to improve motivation. The lack of interaction between the members of the groups requires research to discover how to facilitate members to post and provide social support. This is important as it appears that the social interaction between the interventionist and participants via the text messages results in better outcomes.

## Appendix

Multimedia Appendix 1 E-leaflet provided to participants in Group C.

Multimedia Appendix 2 Questionnaire used to measure change in knowledge at baseline and end of intervention.

Multimedia Appendix 3 Change in the amount of milk intake from baseline to end of intervention (completers only).

Multimedia Appendix 4 Change in calcium intake per day in mg (excluding milk) from baseline to end of intervention (completers only).

Multimedia Appendix 5 Change in secondary outcomes from baseline to end of intervention (completers only).

Multimedia Appendix 6 Process evaluation of the CAN-DO study on frequency of reading posts, messages and interaction.

Multimedia Appendix 7 Quotations illustrating feedback from participants provided through text message replies and qualitative process evaluation (n=106).

Multimedia Appendix 8 CONSORT‐EHEALTH checklist (V 1.6.1).

## Abbreviations

CAN-DO: CAlcium Nutrition-Dietary Opportunities
e-leaflet: electronic leaflet
GEE: general estimating equation
NSW: New South Wales
OR: odds ratio
REDCap: Research Electronic Data Capture
SEIFA: Socio-Economic Indexes For Areas
