# Supplementary figures and images for: Improving Calcium Knowledge and Intake in Young Adults Via Social Media and Text Messages: Randomized Controlled Trial
Source: JMIR Mhealth Uhealth. 2020 Feb 11;8(2):e16499. doi: 10.2196/16499 (PMC7055802; doi:10.2196/16499)

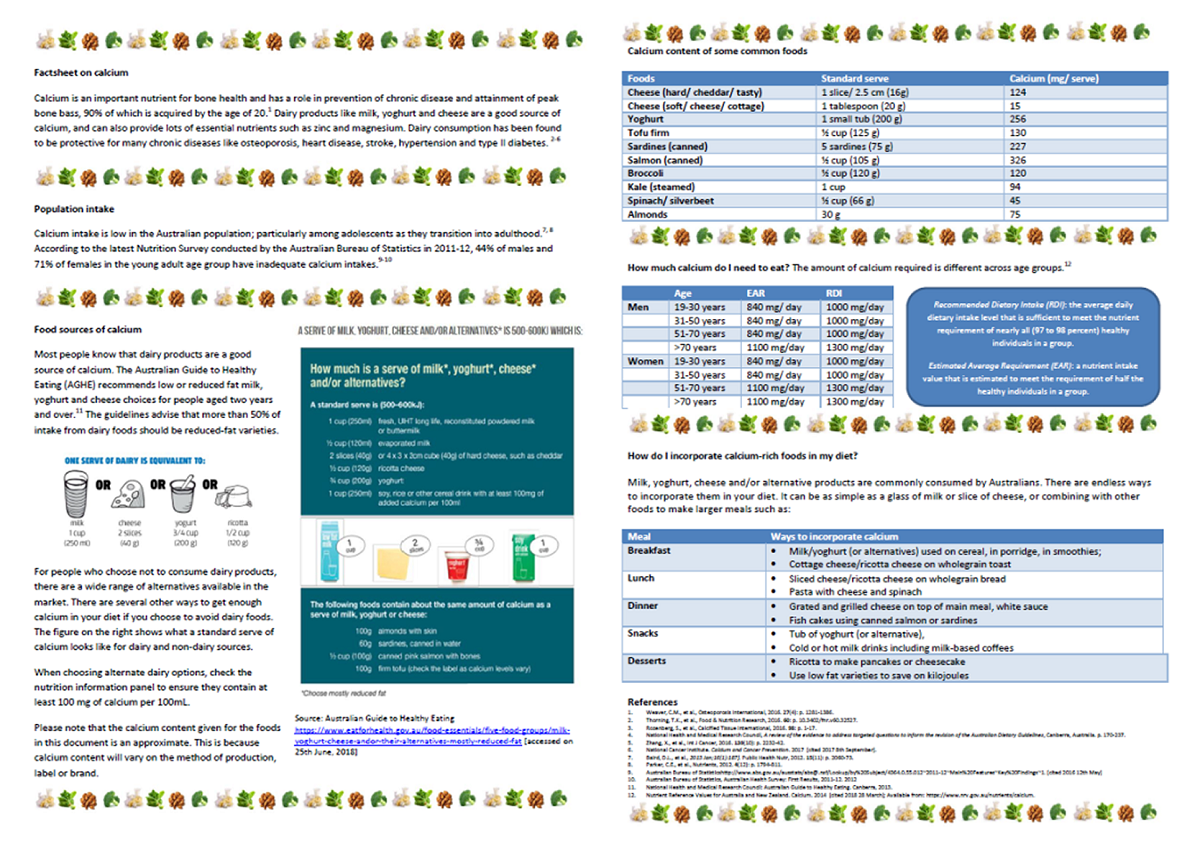

Supplement: Multimedia Appendix 1 [file mhealth_v8i2e16499_app1.png]
