# Supplementary material for: Improving Calcium Knowledge and Intake in Young Adults Via Social Media and Text Messages: Randomized Controlled Trial
Source: JMIR Mhealth Uhealth. 2020 Feb 11;8(2):e16499. doi: 10.2196/16499 (PMC7055802; doi:10.2196/16499)

**Multimedia Appendix 2: questionnaire used to measure change in knowledge at baseline and end of intervention**


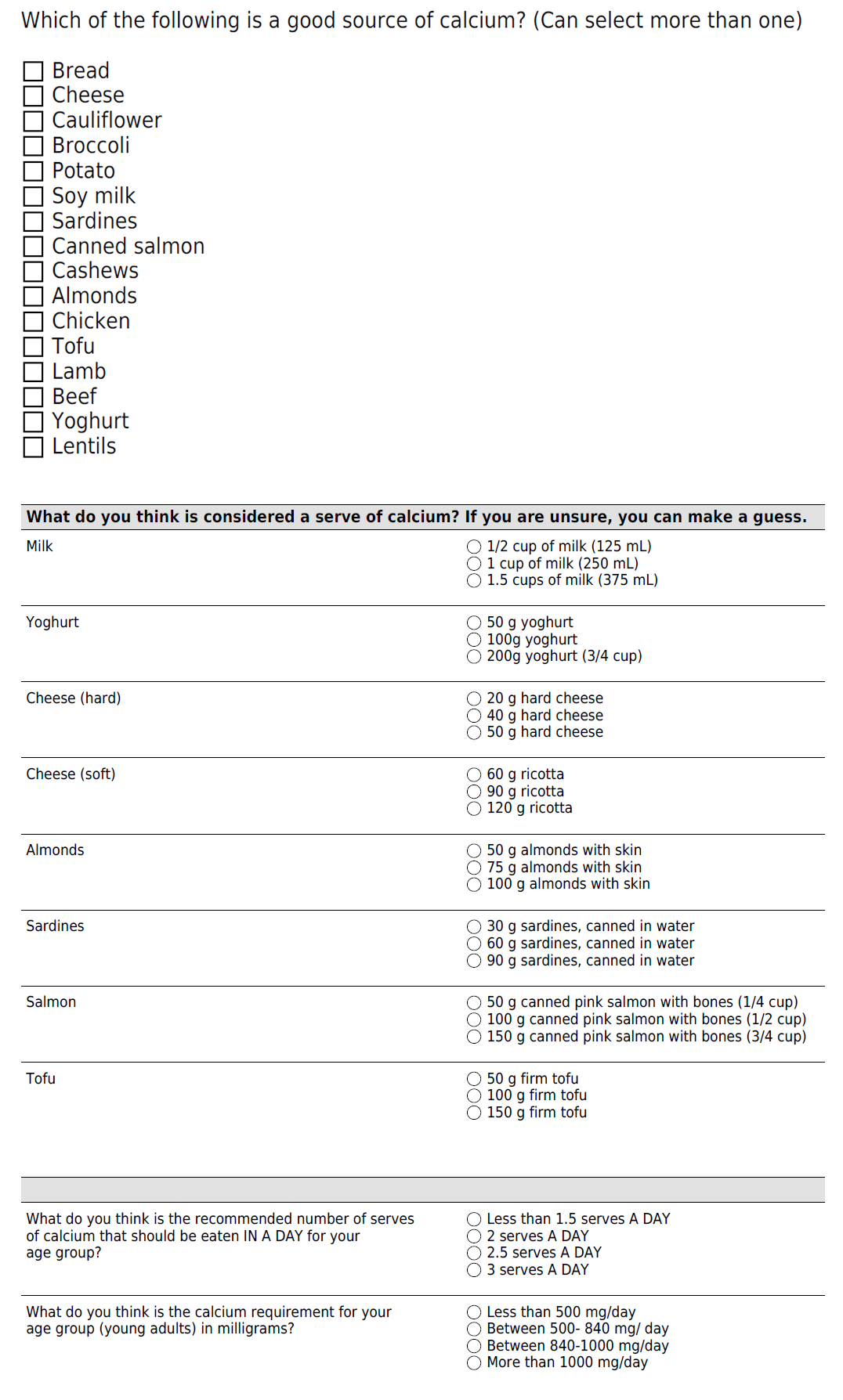

Supplement: Multimedia Appendix 2 [file mhealth_v8i2e16499_app2.docx]
