# Supplementary material for: Improving Calcium Knowledge and Intake in Young Adults Via Social Media and Text Messages: Randomized Controlled Trial
Source: JMIR Mhealth Uhealth. 2020 Feb 11;8(2):e16499. doi: 10.2196/16499 (PMC7055802; doi:10.2196/16499)
Supplement: Multimedia Appendix 3 [file mhealth_v8i2e16499_app3.docx]

**Multimedia Appendix 3: Change in the amount of milk intake from baseline to end of intervention (completers only)**

|  | Baseline milk intake (n) | | | Percent who moved to a higher milk intake category (%) | Percent increase  95% CI (%) | Odds ratio of moving to a higher milk intake category (95% CI) | P value | Overall P value |
| --- | --- | --- | --- | --- | --- | --- | --- | --- |
|  | <125 mL | 125-249 mL | >250 mL |  |  |  |  |  |
| Facebook (n=45) | 17 | 17 | 11 | 56.9 | 44.5, 68.5 | 1.73 (0.60, 5.01) | 0.311 | 0.0180 |
| Facebook plus text (n=42) | 13 | 18 | 11 | 72.0 | 60.7, 81.1 | 4.99 (1.63, 15.28) | 0.005 |  |
| Control (n=49) | 18 | 15 | 16 | 49.3 | 37.6, 61.0 | Reference | - |  |

†Covariates appearing in the logistic regression model have been adjusted for gender, SEIFA, cooking frequency, baseline calcium intake, baseline knowledge, habit, motivation and self-efficacy. The logistic regression model was not adjusted for baseline milk consumption due to everyone in lower category having to stay the same or increase or everyone in the higher category having to stay the same or decrease. This resulted in a zero-cell count for these baseline categories in the respective outcome (0= same or decrease, 1= increase).
