# Supplementary material for: Improving Calcium Knowledge and Intake in Young Adults Via Social Media and Text Messages: Randomized Controlled Trial
Source: JMIR Mhealth Uhealth. 2020 Feb 11;8(2):e16499. doi: 10.2196/16499 (PMC7055802; doi:10.2196/16499)
Supplement: Multimedia Appendix 4 [file mhealth_v8i2e16499_app4.docx]

**Multimedia Appendix 4 Change in calcium intake per day in mg (excluding milk) from baseline to end of intervention (completers only)**

|  | Mean baseline intake (SE) | Mean change (SE) | 95% CI | P value |
| --- | --- | --- | --- | --- |
| Facebook (n=16) | 264.2 (38.5) | 1.6 (35.7) | -70.0, 73.2 | 0.9188 |
| Facebook plus text (n=14) | 234.9 (61.8) | 63.3 (34.9) | -6.7, 133.4 |  |
| Control (n=22) | 256.4 (35.8) | 22.1 (41.3) | -60.8, 104.9 |  |

†Covariates appearing in the linear regression model have been adjusted for gender, SEIFA, cooking frequency, baseline milk intake, baseline calcium intake, baseline knowledge, baseline habit, baseline motivation and baseline self-efficacy.
