# Supplementary material for: Improving Calcium Knowledge and Intake in Young Adults Via Social Media and Text Messages: Randomized Controlled Trial
Source: JMIR Mhealth Uhealth. 2020 Feb 11;8(2):e16499. doi: 10.2196/16499 (PMC7055802; doi:10.2196/16499)
Supplement: Multimedia Appendix 5 [file mhealth_v8i2e16499_app5.docx]

**Multimedia Appendix 5: Change in secondary outcomes from baseline to end of intervention (completers only)**

|  | Facebook (n=45) | | Facebook plus text (n=45) | | Control (n=50) | | P value |
| --- | --- | --- | --- | --- | --- | --- | --- |
| Outcome | Mean Baseline value (SE) | Mean change (95% CI ) | Mean Baseline value (SE) | Mean difference (95% CI) | Mean Baseline value (SE) | Mean difference (95% CI ) |  |
| Habit formation score  (out of 28) | 16.2 (0.8) | 3.4 (1.6, 5.2) | 16.1 (0.9) | 1.1 (-0.5, 2.6) | 16.1 (0.9) | 3.4 (1.9, 4.9) | 0.2238 |
| Overall knowledge score  (out of 18) | 6.9 (0.3)* | 1.6 (0.7, 2.5) | 6.3 (0.3) | 3.0 (1.9, 4.1) | 6.7 (0.3) | 0.26 (-0.7, 1.2) | 0.0397 |
| Motivation score  (out of 16) | 10.5 (0.4) | 1.1 (0.4, 1.8) | 10.5 (0.4)* | 1.4 (0.6, 2.1) | 10.5 (0.3)* | 1.3 (0.5, 2.0) | 0.7805 |
| Self-efficacy  (out of 25) | 19.9 (0.6) | 1.2 (0.1, 2.3) | 19.4 (0.6)* | 1.0 (-0.2, 2.3) | 17.6 (0.7)* | 1.7 (0.3, 3.1) | 0.1320 |

†Covariates appearing in the *linear regression* model have been adjusted for gender, SEIFA, cooking frequency, baseline milk and calcium intake, baseline knowledge, habit, motivation and self-efficacy. * indicates data missing from one participant.
