# Supplementary material for: Improving Calcium Knowledge and Intake in Young Adults Via Social Media and Text Messages: Randomized Controlled Trial
Source: JMIR Mhealth Uhealth. 2020 Feb 11;8(2):e16499. doi: 10.2196/16499 (PMC7055802; doi:10.2196/16499)
Supplement: Multimedia Appendix 6 [file mhealth_v8i2e16499_app6.docx]

**Multimedia Appendix 6: Process evaluation of the CAN-DO study on frequency of reading posts, messages and interaction**

| Questions | Frequency | Facebook (n=46) | Facebook plus text  (n=44) |
| --- | --- | --- | --- |
| How often did you read the text messages? | All the time | Not applicable for this group as they were not sent any text messages | 35 |
|  | More than once a week |  | 7 |
|  | Less than once a week |  | 0 |
|  | Rarely |  | 2 |
|  | I did not receive any text messages |  | 0 |
| How often did you read the Facebook posts? | All the time | 10 | 20 |
|  | More than once a week | 17 | 11 |
|  | Less than once a week | 7 | 7 |
|  | Rarely | 6 | 3 |
|  | I did not see any Facebook posts | 6 | 3 |
| Did you share your own content in the Facebook group? | Yes | 0 | 4 |
|  | No | 37 | 38 |
|  | I was not included in a Facebook group | 9 | 2 |
