# Supplementary material for: Improving Calcium Knowledge and Intake in Young Adults Via Social Media and Text Messages: Randomized Controlled Trial
Source: JMIR Mhealth Uhealth. 2020 Feb 11;8(2):e16499. doi: 10.2196/16499 (PMC7055802; doi:10.2196/16499)
Supplement: Multimedia Appendix 7 [file mhealth_v8i2e16499_app7.docx]

**Multimedia Appendix 7: Quotations illustrating feedback from participants provided through text message replies and qualitative process evaluation (n=106)**

| Themes | Supporting quotes |
| --- | --- |
| Goal setting | - *Goal is two weeks without missing a day, so far on track!* [Female, Facebook plus text] - *Yes I achieved my goals today*. [Female, Facebook plus text] - *Yes, I have achieved my personal goals of having Greek yoghurt/ some sort of milk or calcium added to my breakfast and lunch everyday*. [Female, Facebook plus text] |
| Demonstrates improvement in intake | - *Life has been so busy for me at the moment but I have been remembering to eat yoghurt and that’s nice*. [Female, Facebook plus text] - *...more mindful of my calcium intake and having a little more each day*. [Female, Facebook plus text]. - *I remembered to buy strawberry Yoplait yoghurt and started drinking milk today so that was nice*. [Female, Facebook plus text] - *Yes I have achieved my personal goal of the having Greek yoghurt/ some sort of milk or calcium added into my breakfast and lunch everyday*. [Female, Facebook plus text] - *Already picked up plenty of yoghurt and some cheese this week.* [Female, Facebook plus text] - *I have been consuming approximately 2-3 serves of calcium per day since beginning of this study. It has really opened my eyes to foods which are calcium rich that I was unaware of, such as tofu and some fish*. [Female, Facebook plus text] |
| Ease of use | - *The survey was very easy to use*. [Female, Facebook] - *It didn't feel very interactive*. [Female, Facebook] - *More instructions could have made the study clearer*. [Female, Facebook plus text] - *The program was easy to follow, but I noticed people were confused as to what they were doing, as the amount of participation was variable*. [Male, Facebook plus text] |
| Raised awareness | *For myself, I feel much more knowledgeable in this area than before, as before I wouldn’t even know the recommended intake or which foods are high in calcium*. [Female, Facebook plus text] |
| Feedback on recipes | - *Showing recipes made it so much easier and more fun than just reading listed foods*. [Female, Facebook] - *The recipes were pointless. The picture with the serve info was the only information I cared about*. [Male, Facebook plus text] - *I wasn’t trying the recipes but I thought they were great, planning to try some in the future*. [Female, Facebook plus text] - *The breakfast smoothie idea is now a staple!* [Female, Facebook plus text] - *I loved the recipes and shared them with my family*. [Female, Facebook plus text] |
| Reason for reading text messages | - *The text messages were a good way to be reminded to check my calcium intake.* [Male, Facebook plus text] - *They were relevant and interesting.* [Female, Facebook plus text] - *It was good to have an instant reminder to check my habits or provide ideas*. [Female, Facebook plus text] |
| Reason for not sharing content | - *I don’t like to share on Facebook.* [Female, Facebook] - *I’m reluctant to share information online*. [Female, Facebook] - *I’m more of a passive participant, I did watch some videos but didn’t feel like posting anything*. [Female, Facebook] - *I would have if it was anonymous*. [Female, Facebook plus text] - *I didn’t feel comfortable including my own ideas. I did appreciate when someone else posted though.* [Female, Facebook plus text] |
| Overall feedback and suggestions for improvement | - *I think if it was on another site [ie, not Facebook], I would go on it more often [I rarely use Facebook]. Also, if there was an active chat with all the members talking and posting pictures it would be more fun and interesting.* [Female, Facebook] - *The constant texts and notifications are kind of annoying. Perhaps there could be a less intrusive way of providing the information*. [Female, Facebook plus text] - *Lots of the meals worked and were delicious! I will definitely keep using the app and still follow the page if possible*. [Female, Facebook plus text] - *Thanks for the study, it really helps me realise how much calcium I was lacking and keep me in track with my calcium consumption.* [Female, Facebook plus text] - *Loved all the recipes and all the tips, definitely will increase the calcium in my diet after this!* [Female, Facebook plus text] - *The study has really opened my eyes to calcium rich foods that I didn’t expect to be calcium rich, such as tofu and sardines. The recipes were delicious and easy to make, and a great initiative to encourage myself to increase my calcium*. [Female, Facebook plus text] |
